# Clinical relevance of leukocyte-associated endotoxins measured by semi-automatic synthetic luminescent substrate method

**DOI:** 10.1038/s41598-023-29199-3

**Published:** 2023-02-03

**Authors:** Mari Terayama, Gaku Takahashi, Maria Nonoguchi, Shigenori Kan, Koichi Hoshikawa, Katsuya Inada, Tomohiko Mase

**Affiliations:** Department of Critical Care, Disaster and General Medicine, School of Medicine, Iwate Medical University, 2-1-1 Idaidori, Yahaba Town, Iwate 028-3695 Japan

**Keywords:** Diagnostic markers, Predictive markers, Biomarkers

## Abstract

A newly developed semi-automatic synthetic luminescence substrate (SALS) method for measuring endotoxin was compared with the existing turbidimetric kinetic assay (TKA) using leukocyte-rich plasma to verify its usefulness. As a result, the endotoxin levels by this method were higher than that by the existing assay in most specimens, and the time required for measurement was much shorter. In addition, the leukocyte-rich plasma endotoxin level minus the plasma endotoxin levels were named leukocyte-associated endotoxin, and these levels per one leukocyte were compared. As a result, those levels were highly correlated with the endotoxin measurement levels of leukocyte-rich plasma. The correlation coefficient of SALS method was superior to the existing TKA method, the endotoxin level by this method may be close to true endotoxin levels.

## Introduction

Endotoxins are cell wall component of gram-negative bacteria, and its chemical entity is lipopolysaccharide (LPS), and have a variety of biological activities. The pathophysiological role of endotoxins in sepsis and septic shock should be of crucially concern. Endotoxins in the blood could be measured by the Limulus test relying on a gelation reaction in horseshoe crab amebocyte lysate^1^. Then, as a measurement method using this principle, quantitative synthetic chromogenic substrate method^2,3^ and turbidimetric kinetic assay (TKA)^4^ were developed. Endotoxin-specific assays designed to not react with glucan are has been used in Japan^5,6^. The TKA has been approved for reimbursement by Japanese National Health Insurance as a plasma endotoxin assay. This assay detects an increase in turbidity, and has excellent lower detection limit. However, its longer measuring time, more than 2 h, and low detective sensitivity are considerably problems.

Noda et al., developed a synthetic luminescent substrate method, using a high intensity-luminescence mutant firefly luciferase whose luminescence intensity is more than 10 times as intense as the wild type^7,8^, with the aim of developing a rapid and highly sensitive endotoxin assay. This method uses a synthetic luminescent substrate (benzoyl-Leu-Gly-Arg-aminoluciferin) for the coagulation enzyme in amebocyte lysate. The released luciferin is luminescent by the luciferase in the presence of ATP. Onodera et al. suggested that this method could be used to the quantitative measurement of endotoxin in human blood^9^. However, this method requires rapid hand technique as luminescence occurs immediately upon the addition of luciferase. Recently, a semi-automated luminescent substrate method (SALS) was developed. However, this device was developed to measure endotoxin in saline and distilled water, and has not been studied for measuring human blood samples. When measuring human blood samples, the protein in the blood has the greatest effect on the measured levels. In the TKA method using PRP samples, studies have also been conducted on measurement inhibitors. The current method recommends diluting the sample tenfold, heating the sample at 75 °C for 5 min, and then cooling it on ice for 5 min. First, we examined the effect of proteins in the blood on this device. We also reported that detection sensitivity is improved by using leukocyte-rich plasma (LRP) obtained by the dextran warming method^10^. This study uses these new methods to measure endotoxins.

Most endotoxins are thought to exist in the blood in a state of being taken up by leukocytes. However, the existing measurement method only measured endotoxin in plasma, so it is possible that the detection sensitivity was low. It has already been reported that a SALS using LRP as the measurement sample dramatically improved the measurement accuracy, but the effect of leukocyte counts on the measurement levels have not yet been investigated. In this study, the LRP endotoxin level minus the plasma endotoxin level was named leukocyte-associated endotoxin, and the relationship with leukocyte counts were also examined in detail.

## Materials and methods

### Pretreatment solution for plasma

A pretreatment solution for plasma endotoxin assay (0.02% Triton X-100 solution, FUJIFILM Wako Pure Chemical Corporation, Osaka, Japan) was used^6^.

### Limulus amebocyte lysate (LAL)

Lyophilized LAL; Endotoxin Single Test WAKO (FUJIFILM Wako) was used. This reagent is specific for endotoxin by adding glucan ^6^.

### Dextran T500

Six %(W/V) Dextran T-500 (Pharmacosmo, Holbaek, Denmark), an average molecular weight of 500,000 Da, was prepared in physiological saline for injection (Otsuka Pharmaceutical Co., Ltd., Naruto, Japan) and autoclaved it at 121 °C for 90 min to ensuring to be endotoxin-free. In other words, no gelation occurred even after the maximum measurement time (200 min) of incubation in TKA (Endotoxin content is calculated to be less than 0.05 pg/ml).

### Blood specimens

Blood samples were obtained from 28 patients and 8 healthy individuals. Patients who were transported to the Iwate Medical University Advanced Critical Care Center and were suspected of having a bacterial infection were approached for enrollment in this study. Among them, 22 patients were gram-negative bacterial infections by the results of bacterial culture of obtained specimens, and others were having gram-positive infections. The results of eight healthy individuals did not mentioned here because the endotoxin levels were too lower to evaluate. The “International Consensus Definition of Sepsis and Septic Shock, 3rd Edition (Sepsis-3)” was used to identify bacterial infection, sepsis, and septic shock^11^.

### Preparing plasma

Heparinized blood was centrifuged at 3000 rpm for 40 s with a bench-top centrifuge and obtained plasma^12^.

### Preparing LRP by LRP37 method

Heparinized blood was collected and equilibrated at 37 °C for about 10 min. Next, 800 µL of the blood and 400 µL of the 37 °C-equilibrated 6% dextran T500 were mixed and incubated at 37 °C about 15 to 20 min until almost clear upper layer was obtained^10^. The upper layer was collected as the LRP, whereas the lower layer was red blood cells layer aggregated by dextran.

### Sample treatment for endotoxin assay

Plasma and LRP were diluted 10-times with the pretreatment solution. In the case of LRP, LRP was mixed vigorously with a vortex mixer (MS-3, IKA Japan, Osaka, Japan) to ensure that leukocytes were destroyed^10^. Then that was heated at 70 °C for 10 min to inactivate the interfering factors^5^. The sample was ice-cooled and then used for measurement by TKA. In SALS, the sample was further diluted with water as described below.

### Turbidimetric kinetic assay (TKA)^6^

LAL was dissolved with 200 µL of the pretreated plasma or LRP, and placed the test tube to Toxinometer ET-5500 (FUJIFILM Wako) and incubated until 200 min. The transmittance at the start was set at 100%, and the gelation time was defined as the reaction time required for the percent transmittance of a reaction mixture to reach 92%. We then calculated the amount of endotoxin in the sample from the standard curve that had been plotted by using *E.coli* O111:B4 LPS.

### SALS

**Figure 1:**
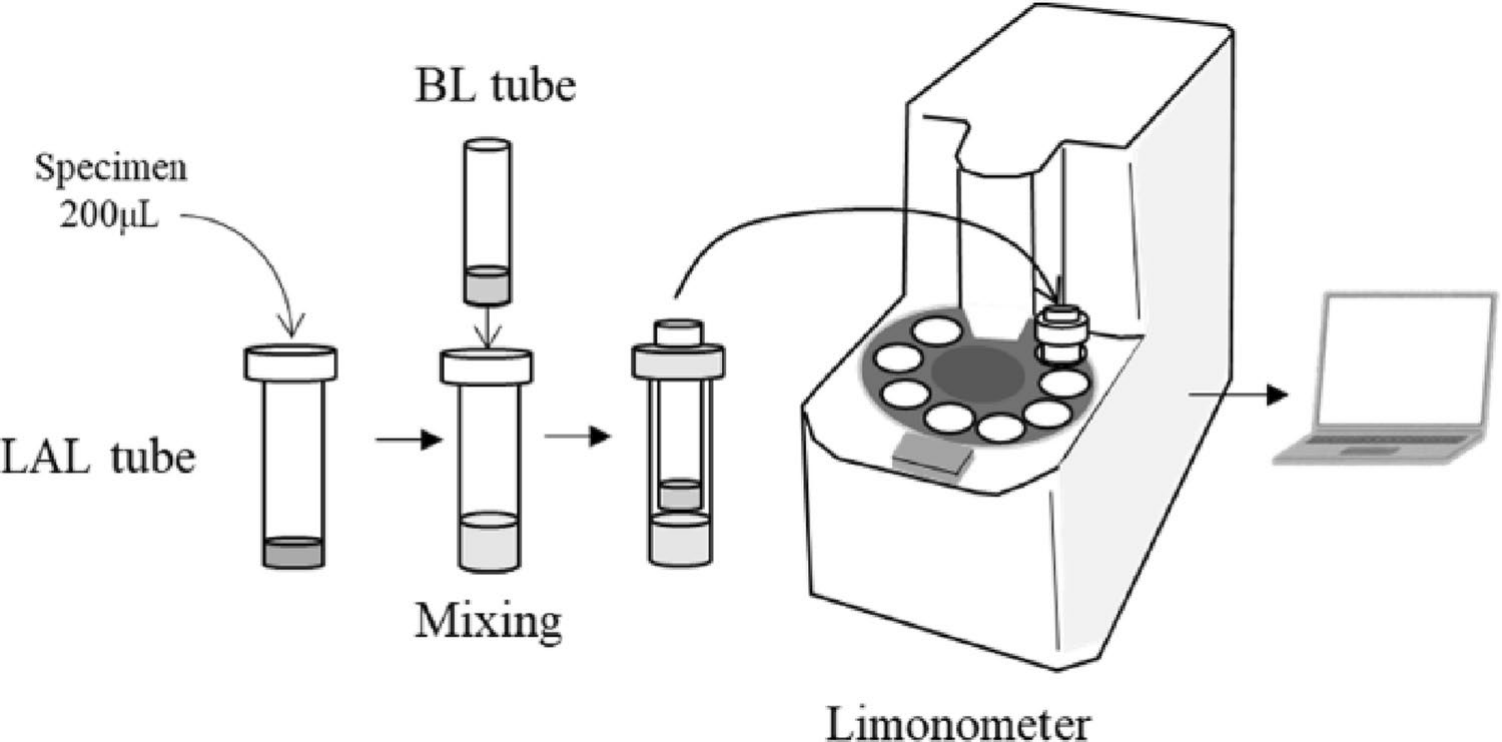
Illustration of the SLAS method (Luminutes-ET). The LAL tube containing specimen and LAL and BL tubes containing the substrate, ATP, and Luciferase are inserted into the well of the instrument. The LAL and specimen are mixed and heated to 37 °C for 19 min. Then the substrate, ATP, and luciferase in the BL tube are then automatically injected into the LAL and specimen mixture and the luminescence is immediately measured.

SALS developed for the endotoxin detection of dialysate in blood dialysis, by DKK-TOA Corporation (Tokyo, Japan) was used. The system consists of LAL tube, bioluminescence (BL) tube and a specially made luminometer **Luminutes**^**®**^**-ET** connected with Windows PC. The LAL tube contains the LAL reagent. The BL tube, which is smaller in diameter than the LAL tube, contains ATP, luciferase and synthetic luminescent substrate Benzoyl-Leu-Gly-Arg-aminolluciferin, which are automatically added to the reaction mixture in the LAL tube, when the reaction is completed. Briefly, 200 µL of pretreated plasma or LRP, which was further diluted as described below, was added to LAL tube, then the BL tube was inserted into the LAL tube, and the two combined tubes were stirred for 5 s. Here the sample and LAL were mixed. Next, the combined tubes were set in the luminometer and incubated at 37 °C for 19 min. At the end of incubation**,** ATP, luciferase and the substrate were automatically poured into the mixture and the bioluminescence reaction occurred immediately. The amount of luminescence was measured, and endotoxin content was calculated using software incorporating a calibration curve. High intensity-luminescence mutant firefly luciferase was isolated by DKK-TOA Corporation for this assay. Eendotoxin-specific LAL (FUJIFILM WAKO) was used in this assay. (Fig. 1).

### Endotoxin level integrity in both methods

In TKA, the amount of endotoxin was expressed in pg/mL usually, but here, it is expressed in endotoxin units as in SALS. In this case, 1 pg was expressed as 7 mEU by the company’s information. To obtain the LRP, half the volume of dextran was added to one volume of blood, therefore, the LRP endotoxin level was corrected using the following formula to compare with plasma endotoxin. The LRP endotoxin value (pg/mL) was obtained by multiplying the actual value obtained with the LRP method by the coefficient ([A + dextran amount]/A), wherein A = blood volume−blood volume × hematocrit value/100. For example, if the blood volume and hematocrit value are 800 μL and 30, respectively, A is calculated as 560 (800–800 × 0.3). As the dextran volume is 400 μL, a multiplying factor of 1.71 is obtained by (560 + 400)/560^10^.

### Definition of leukocyte-associated endotoxin

Leukocyte-associated endotoxin means LRP endotoxin level minus plasma endotoxin level. This is because LRP endotoxin levels are considered as the sum of plasma and leukocyte-associated endotoxin values.

### Statistical analysis

Spearman's rank correlation coefficient test was performed using SPSS software. Significant differences were regarded as those with p-values of less than 0.05.

### Informed consent

Written informed consent was obtained from patients or the patients’ family members.

### Ethical approval

Ethical approval for the study (Ethical Committee No. H29-174) was provided by the Ethical Committee of Iwate Medical University. All research was conducted in line with the Declaration of Helsinki.

## Results

### Effect of plasma proteins and the detection of dilution factor in SALS

**Figure 2:**
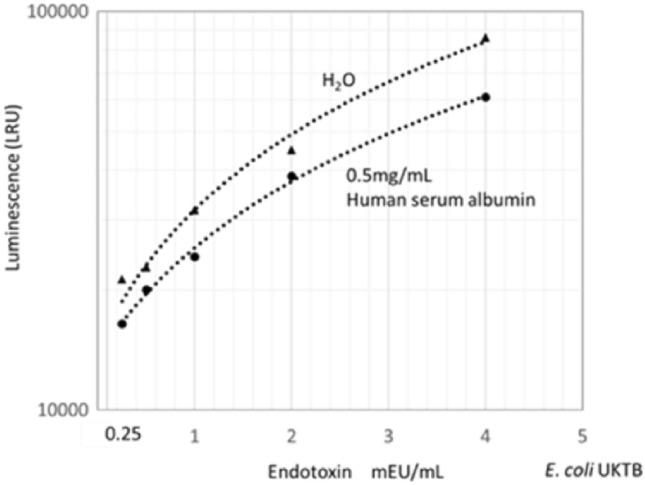
Calibration curves for measuring endotoxin. Relationship between LPS (UKTB) dissolved in saline or 0.5% human serum albumin and bioluminescence measured by SALS was depicted.

As plasma proteins suggested to inhibit bioluminescence as Onodera et al. reported^9^, so in the SALS, LRP was diluted with water to minimalize the effect of the proteins. Total protein in plasmas used in this study was about 49 mg/mL. Furthermore, specimens were initially diluted about 1.71 times due to the dextran addition as the specimens’ average hematocrit (30%) and further diluted 10 times with pre-treatment solution as for TKA. It was further diluted fivefold with water to minimize the effect of protein (final 85-fold) as a sample for SALS. Thus, the protein content of the diluted LRP was finally about 0.58 mg/mL. SALS was influenced by the addition of more than 0.1 mg/mL of human serum albumin solution (HSA) (data not shown). The calibration curve using UKTB LPS shown in Fig. 2 indicates that approximately 25% reduction of LPS-induced bioluminescence in the presence of 0.5 mg/mL of HSA.

### Endotoxin levels in TKA and SALS

**Figure 3:**
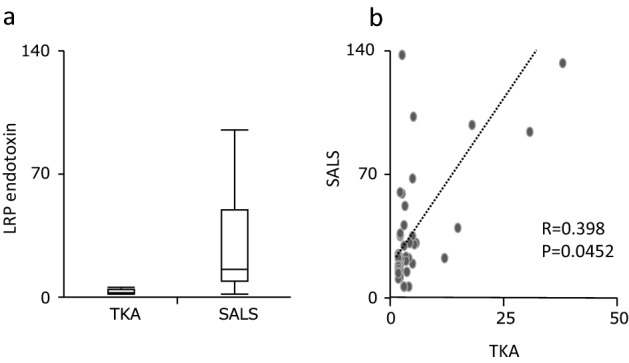
Correlation between LRP endotoxin contents measured by SALS and TKA. Endotoxin contents in LRP measured SALS and TKA by were shown (**a**), and the relationship between endotoxin contents measured by SALS and TKA were shown (**b**).

As shown in Fig. 3, LRP endotoxin levels measured by SALS tended to be higher than those measured by TKA in most cases. A significant correlation was also observed between both measurements.

### Relationship between endotoxin and WBC counts

**Figure 4:**
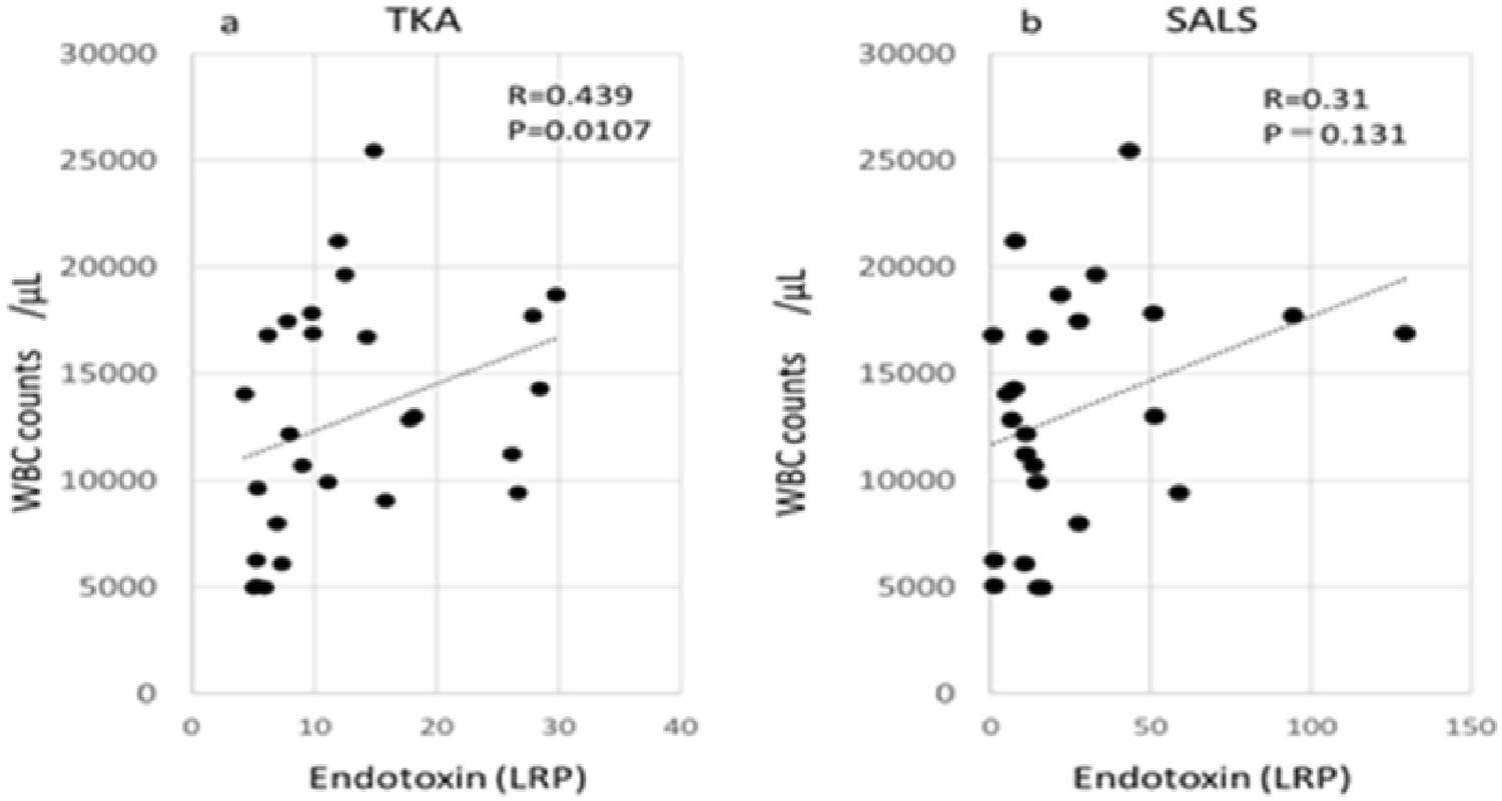
Correlations between LRP endotoxin and white blood cells (WBC) counts/μL.

As shown in Fig. 4, LRP endotoxin levels tended to increase with increasing white blood cell counts. In particular, it was suggested that the TKA method may be more strongly affected by the white blood cell count.

### Relationship between LRP endotoxin and leukocyte-associated endotoxin in both methods

**Figure 5:**
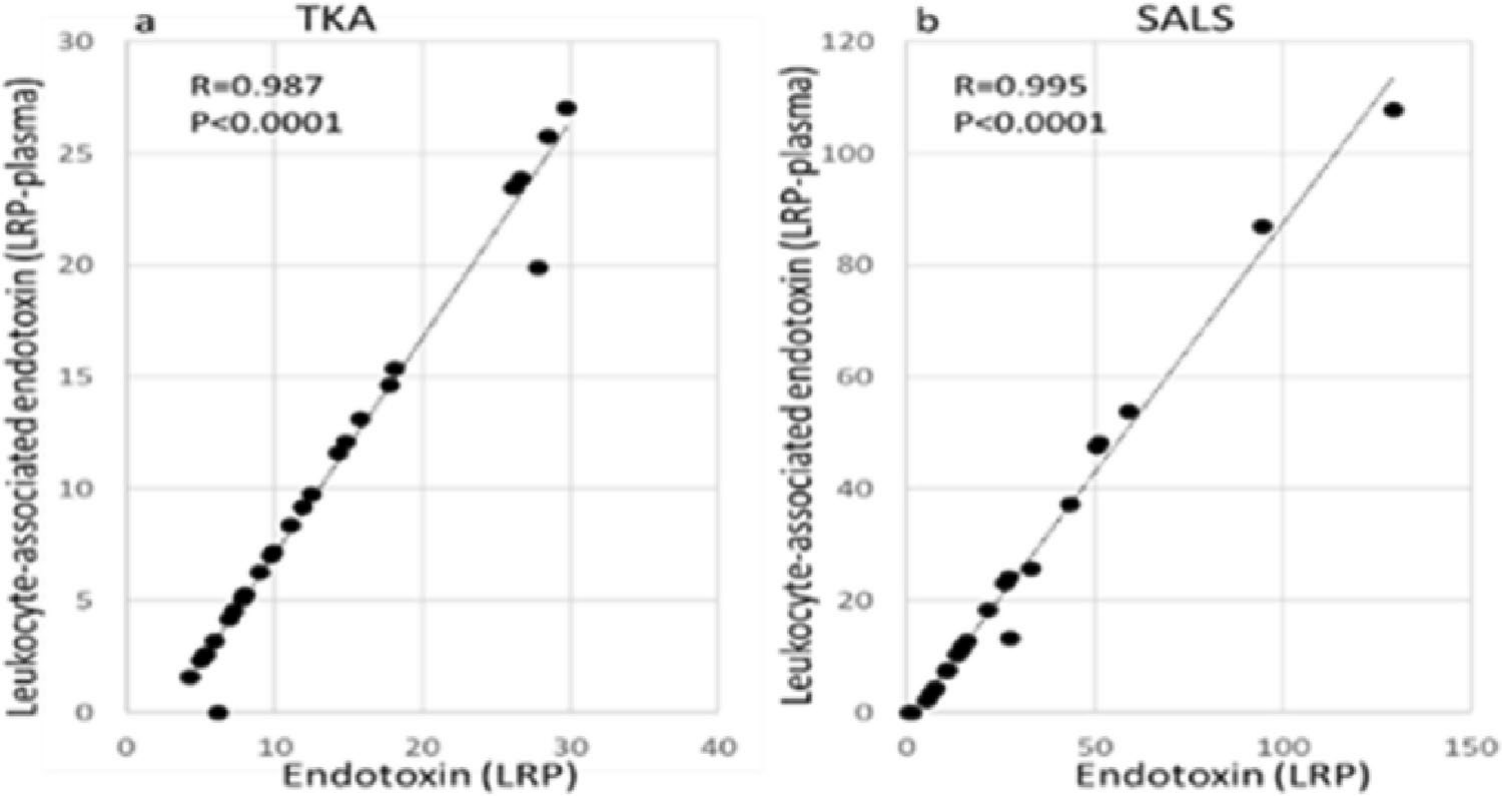
Correlations between LRP endotoxin contents and leukocyte-associated endotoxin contents.

In this study, the LRP endotoxin level minus the plasma endotoxin level was named leukocyte-associated endotoxin. As shown in Fig. 5, the value was found to be strongly correlated with the LRP endotoxin level.

### Leukocyte-associated endotoxin level per one leukocyte

**Figure 6:**
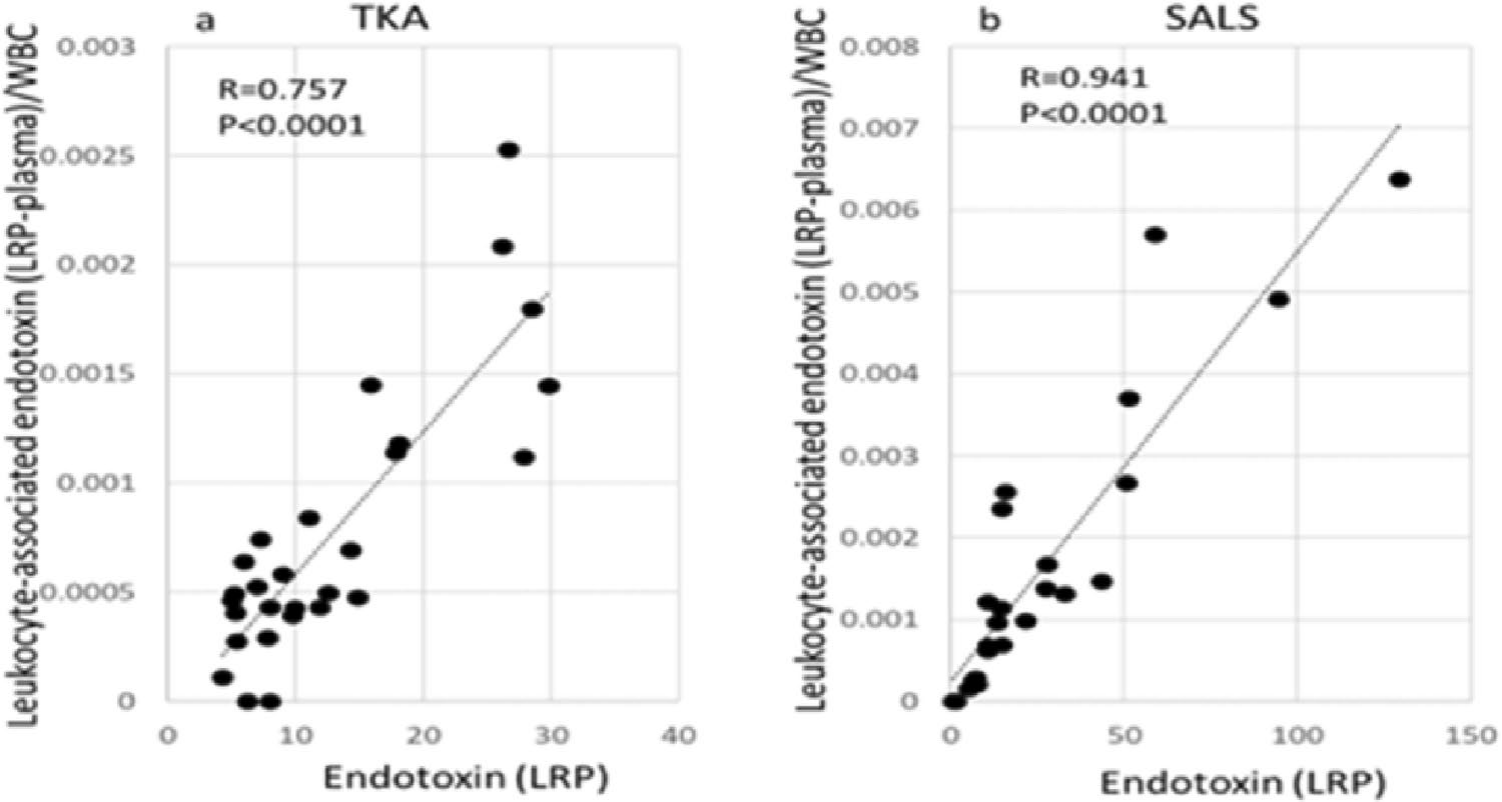
Correlations between LRP endotoxin and leukocyte-associated endotoxin/WBC.

As shown in Fig. 6, the leukocyte-associated endotoxin level per leukocyte correlated with the LRP endotoxin level, and a particularly strong correlation was observed in SALS.

## Discussion

SALS was originally developed to measure dialysate endotoxin without considering the effects of proteins, thus its compatibility with human plasma has not been known. The result of the present study showed that the inhibitory effect of plasma proteins to the luminescence reaction was evident: 85-fold diluted plasma still had the inhibitory effect. Nevertheless, the results by SALS showed higher endotoxin levels than TKA. This may be due to the fact that the matrix effect exhibited by the plasma was attenuated by dilution rather than the property of SALS: SALS measures the luminescence reaction induced by luciferase, whereas TKA captures the turbidity increment caused by coagulase production by coagulase. The presence of some samples with low levels of endotoxin in SALS cannot be ignored. Protein levels in these samples were not higher than in other samples. This issue should be explained in the future if SALS is to claim its superiority. Thus, SALS could be used as a more reliable assay for human blood-derived endotoxin if it minimizes the effect of protein concentration or improves detection sensitivity. The advantage of this method is that it takes only about 20 min to get results, which is much less time than the TKA method. In addition, Nonoguchi et al. ^13^ has revealed that the endotoxin levels measured by SALS well correlated with the severity score of sepsis, i.e. SOFA (sequential organ failure assessment) score or APACHE (acute physiology and chronic health evaluation) II score in sepsis. From these points, we believe that SALS has sufficient potential for clinical application in the future.

The method of using LRP instead of plasma as the target of endotoxin measurement has already been reported by Kan et al.^14^, and a method of collecting LRP at 37 °C using dextran as an aggregating agent has recently been devised by us (LRP37 method)^10^. Samples obtained by this method were used for measurement of this study.The method to obtain LRP uses the aggregating agent dextran under 1 × *g* conditions to remove erythrocytes which interfere the limulus test, and leukocytes are still suspended in plasma. The causative bacterium in LRP probably remains without sedimentation. In LRP, endotoxins and bacteria are thought to be bound to leukocytes or taken up by them to activate cells. In contrast, plasma is obtained by centrifugation of all blood cell components, including erythrocytes and leukocytes, and even under minimal centrifugation conditions that remove erythrocytes, many bacteria will probably be sedimented^12^. In this study, the leukocyte-rich plasma endotoxin level minus the plasma endotoxin level was named leukocyte-associated endotoxin. This value is considered to be purely the value of endotoxin taken up by leukocytes. There is a strong correlation between LRP endotoxin and leukocyte-associated endotoxin, and thus leukocyte-associated endotoxins seem to be superior to the plasma endotoxin in sepsis. Since it is speculated that most endotoxins are taken up by leukocytes and are present, it is undeniable that endotoxin measurements may be affected by leukocyte counts. Even in the cases we examined, we found that there was some association between peripheral white blood cell count and endotoxin levels. Therefore, in order to offset this relationship, the measured value was divided by the number of peripheral white blood cells to calculate the endotoxin value per one leukocyte. Interestingly, that value also correlated very strongly with the LRP endotoxin level. The improved LRP is used as the measurement sample, SALS is used as the measurement instrument, and the leukocyte-associated endotoxin level bound per one leukocyte is calculated from the measured value and the number of leukocytes. As far as we can think of, these may be the ultimate measurement method.

Plasma endotoxin measurement has been performed in the current clinical setting, its clinical importance has been recently underestimated, but these new measurement methods might restore the clinical evaluation of endotoxin measurement again.

## Conclusion

By using LRP instead of plasma in measuring endotoxin levels in human blood, the measurement accuracy has improved dramatically. Also, by using SALS for the measuring machine, the measuring time has been greatly reduced. Since most endotoxins may be taken up and present in leukocytes, leukocyte-related endotoxin levels and endotoxin levels per one leukocyte may be close to true endotoxin levels.

## Data Availability

The datasets generated during and/or analysed during the current study are available from the corresponding author on reasonable request.
