# Author Correction: Clinical relevance of leukocyte-associated endotoxins measured by semi-automatic synthetic luminescent substrate method

**DOI:** 10.1038/s41598-023-31267-7

**Published:** 2023-03-14

**Authors:** Mari Terayama, Gaku Takahashi, Maria Nonoguchi, Shigenori Kan, Koichi Hoshikawa, Katsuya Inada, Tomohiko Mase

**Affiliations:** Department of Critical Care, Disaster and General Medicine, School of Medicine, Iwate Medical University, 2-1-1 Idaidori , Yahaba Town, Iwate 028-3695 Japan

Correction to: *Scientific Reports* 10.1038/s41598-023-29199-3, published online 03 February 2023

In the original version of the Article, Figure 3 was a duplication of Figure 5.

The original Figure 3 and accompanying legend appear below.

**Figure 3:**
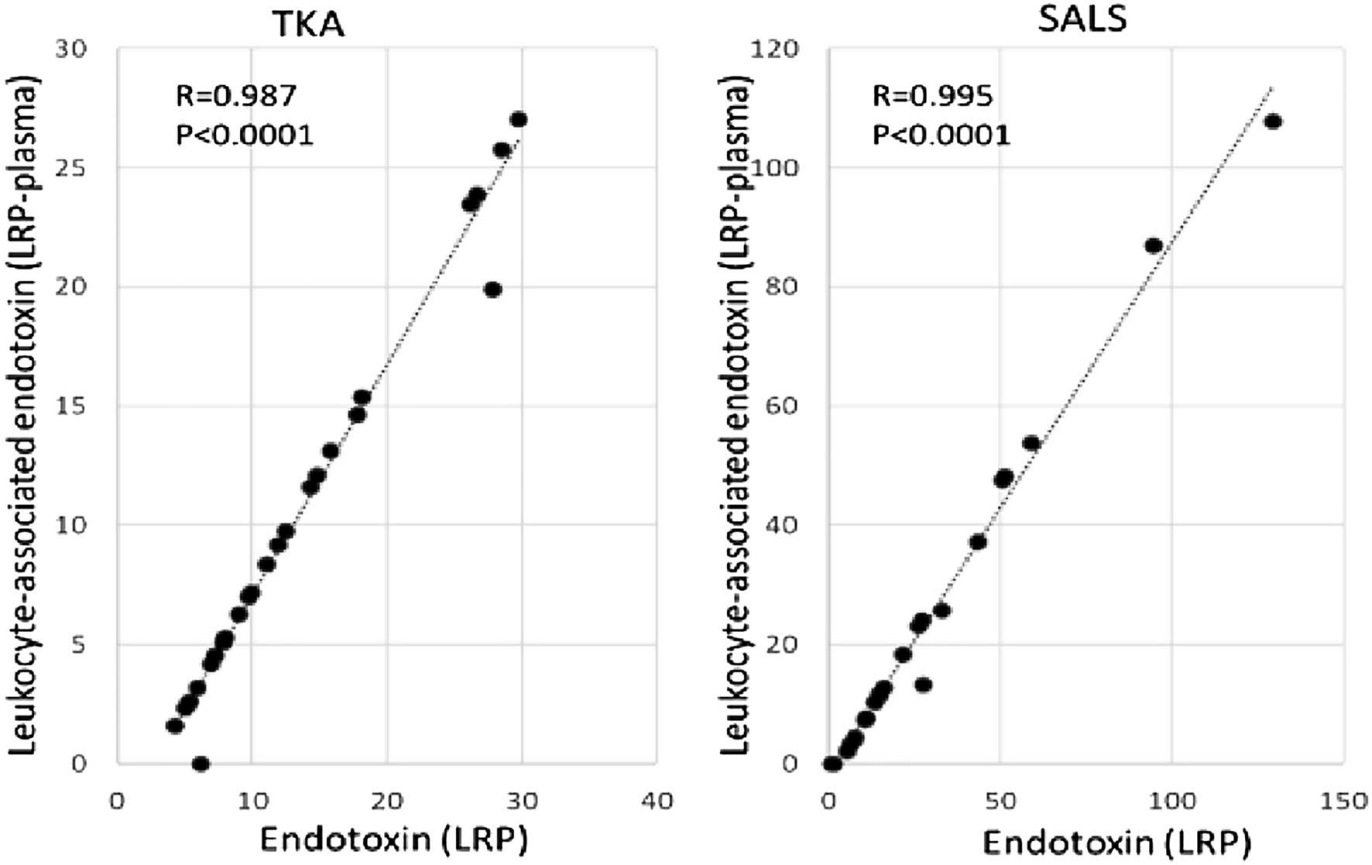
Correlation between LRP endotoxin contents measured by SALS and TKA. Endotoxin contents in LRP measured SALS and TKA by were shown (**a**), and the relationship between endotoxin contents measured by SALS and TKA were shown (**b**).

The original Article has been corrected.

